# Encapsulation of Olive (*Olea europaea* L.) Pruning Waste Particles by Supercritical CO_2_ Technology

**DOI:** 10.3390/foods13060905

**Published:** 2024-03-16

**Authors:** Antonio Montes, Diego Valor, Ignacio García-Casas, Ana Sánchez, Clara Pereyra

**Affiliations:** Department of Chemical Engineering and Food Technology, Faculty of Sciences, Wine and Agrifood Research Institute (IVAGRO), University of Cadiz, 11510 Cadiz, Spain; antonio.montes@uca.es (A.M.); ignacio.casas@uca.es (I.G.-C.); anamsanchezb01@gmail.com (A.S.); clara.pereyra@uca.es (C.P.)

**Keywords:** supercritical, encapsulation, carbon dioxide, antioxidant, *Olea europaea* L.

## Abstract

Olive leaves (*Olea europaea* L.) contain a multitude of bioactive compounds such as sterols, carotenes, triterpenic alcohols and phenolic compounds. These compounds have been shown to exhibit antiviral, antioxidant, candida-growth-inhibitory, anticancer, antifungal, anti-inflammatory and antibacterial activities. In this sense, submicron particles from olive leaves with antioxidant activity were precipitated by supercritical antisolvent extraction in a previous work. Moreover, encapsulation enables the delayed release of compounds and avoids the first-step effect in medical therapies. Therefore, this work focused on encapsulation of particles with a certain antioxidant capacity from olive pruning waste using supercritical technology. A variety of experiments were carried out to test how the different operating variables (pressure, temperature and extract–polymer ratio) affect. Morphology was analyzed by SEM microscopy, obtaining encapsulates between 1 and 5 microns in size. The antioxidant capacity was determined by the DPPH assay, with most of the encapsulates having AAI values between 0.5 and 1 (moderate antioxidant capacity). An increase in polyphenol content was observed in the 1:3 ratio tests. The release of the compounds in gastric simulated medium was retarded by the polymeric encapsulation, while in intestinal fluid, the solubility was improved compared to the unencapsulated particles. Overall, the supercritical encapsulation process for the natural extract of olive pruning residues has proven to be effective in obtaining antioxidant particles with different release profiles.

## 1. Introduction

Recently, olive leaves have begun to gain special consideration due to the beneficial properties they contain, which can be focused on in several fields, such as food and biomedicine [1]. The olive tree is an oleaginous crop of the *Oleaceae*, which is native to the Mediterranean coast. In European and Mediterranean countries, olive leaf extract is used in natural remedies. Iranians use olive leaves to treat wounds and skin diseases [2]. More than 200 chemical compounds are found in the olive plant, including stearols, carotenes, triterpene alcohols and phenolic compounds. Among the most abundant triterpenes are oleanolic acid, erythrodiol, maslinic acid and uvaol [3]. There are at least 30 phenolic compounds found in olives, including oleuropein and elecanthal [2].

Polyphenols are a major source of antioxidants for humans. They are derived from plants and are consumed in the diet in the form of fruits, vegetables, spices and herbs. Intake of polyphenols varies among different cultures and ethnic groups, and within the same geographic location [4]. The main phenolic components of olive leaves are hydroxytyrosol, tyrosol and oleuropein. They are believed to be responsible for the pharmacological effects of olive leaves [1]. Olive leaf extract has been shown to have antiviral, antioxidant, candida-growth-inhibitory, anticancer, antifungal, anti-inflammatory and antibacterial activities [3].

In addition, it was recently confirmed in a study that olive leaf extract has a strong antiviral impact against hepatitis virus, herpes mononucleosis, bovine rhinovirus, canine parvovirus, rotavirus, respiratory syncytial virus, parainfluenza virus type 3 and feline leukemia virus. Olive phenols have also been shown to have protective effects against reoxygenation of brain hypoxia and ischemia [3]. The efficacy of olive leaf extract in curing herpes simplex labialis has also been investigated [1]. Herpes simplex virus (HSV) is resistant to drugs and also has side effects against them, which has attracted the attention of researchers. Olive extract has antiviral effects that can treat HSV1.

Olive extract products have been shown to have great therapeutic potential in cancer. Much of the existing research has focused on the use of cell culture models of disease, demonstrating that olive extracts and the compounds within these extracts have efficacy in a variety of in vitro and in vivo cancer models [5]. Therefore, the effect of olive leaf extract on the human lymphoblastic leukemia cell line Jurkat was analyzed [6]. The results reported a 78% inhibition of Jurkat cell proliferation, thus observing a significant decrease in viability. The efficacy of olive leaf extract has also been demonstrated in a pancreatic cancer cell line (MiaPACa-2). At concentrations of 200 μg/mL, olive leaf extract was able to reduce cell viability up to 27%. Juan et al. observed the effect of maslinic and oleanolic acid on HT-29 colon cancer cells. These compounds were examined for their effect on proliferation, necrosis and apoptosis. It was concluded that they altered the expression of cell cycle regulatory proteins in an unconscious manner in different types of cancer cell lines [7].

There are a multitude of techniques to extract the active substance from waste, such as soxhlet extraction, and extraction by maceration and/or hydrodistillation [8]. However, conventional methods usually have drawbacks such as long extraction times, the use of high-purity solvents which are quite expensive, the evaporation of solvents in significant quantities and, in the case of thermolabile substances, thermal decomposition [8,9]. These drawbacks do not generally occur in other non-conventional extraction techniques as supercritical extraction. This technique can be referred to as a “green technique” because it meets the standards set by the U.S. Environmental Protection Agency [8]. These include less hazardous chemical synthesis, safer chemical design, safe solvent auxiliaries, design for energy efficiency, use of renewable raw materials, reduction of by-products, catalysis, design to avoid degradation, atomic economy and time analysis for pollution prevention and inherently safer chemistry for accident prevention. Among high-pressure extraction methods, the high-pressure solvent extraction methods of pressurized liquid extraction (PLE), where CO_2_ is not used, and enhanced solvent extraction (ESE), using a mixture of CO_2_ and solvent, often offer better results. In the present work, an enhanced solvent extraction process was carried out using supercritical fluids. Carbon dioxide was used as solvent and ethanol as co-solvent at 50:50 *w*/*w*. This mixture helps to obtain better extraction efficiency than using only the organic solvent, allowing us to obtain extracts with high concentrations of olive leaf polyphenols [10].

On the other hand, in recent years, nanotechnologies have been applied to human health with promising results, especially in the field of cancer treatment [11]. The advantages of nanoparticles are many, including the ability to cross biological barriers and improved bioavailability [12,13]. In this sense, Song et al. [14] produced Ag/attapulgite nanocomposites using olive leaf extract with a multitude of biological properties, such as antibacterial, antidiabetic, antitumor and wound healing enhancement. It was found in this study that these particles were size-dependent, i.e., smaller particles have greater antibacterial activity. Bocarando-Chacon et al. [15] obtained nanoparticles from *Opuntia ficus-indica* extract. This plant has a very beneficial point, which is that it does not require a lot of water or special treatments for its care.

Moreover, potent antioxidant submicron particles from grape residues [16], olive [17] and eucalyptus leaves [18] have been produced by supercritical antisolvent extraction (SAE). High antioxidant spherical nanoparticles based on polyphenols from mango [19] and orange [20] leaves were also successfully precipitated by this technique. They could be used in clinical treatments, biomedical devices, pharmaceuticals, dressings, food, coatings and other fields.

However, the use of nanoparticles directly is not recommended. In this sense, biodegradable and biocompatible polymers are widely used to encapsulate an active substance to protect it from degradation from the moment of administration to the patient until it reaches the target organ, and to provide a prolonged release of the component of interest. In addition, these polymers also show good potential for surface modification and functionalization with different ligands, provide excellent pharmacokinetic control and are suitable for encapsulation and delivery of a large number of therapeutic agents [13]. Thus, Machado et al. [21] prepared submicron particle composites from grape residue extract and polyvinylpyrrolidone (PVP) by the SAE process. Guaman-Balcazar et al. also encapsulated mango leaf particles with the same polymer PVP [22]. Rosemary antioxidants were encapsulated [23] with poloxamers, and Santana and Meireles achieved the co-precipitation of turmeric extracts using polyethylene glycol [24].

Pluronic F-127 is a polymer composed of propylene oxide for the hydrophobic section in the centre and ethylene oxide for the hydrophilic sections on both sides. Furthermore, it is suitable for controlled release, drug delivery, tissue engineering and gene therapy due to its unique thermo-reversible micellization around body temperature [25,26]. It is one of the most widely used sterically stabilizing polymers in micro/nanostructured drug formulations due to its commercial availability and low cost [27]. In the present work, olive pruning waste extract is used for its subsequent encapsulation with the Pluronic F-127 polymer by the SAE technique. This allows us to obtain Pluronic F-127-olive extract microparticles for possible treatment as a drug system formulation.

## 2. Materials and Methods

### 2.1. Chemicals

Pluronic F-127, 2,2-Diphenyl-1-picrylhydrazyl (DPPH), Folin–Ciocâlteu reagent and gallic acid were purchased from Sigma-Aldrich (Steinheim, Germany). Absolute ethanol, sodium carbonate, sodium chloride, monobasic phosphate and concentrated hydrochloric acid were purchased from PanReac AppliChem (Barcelona, Spain). CO_2_ with a maximum purity of 99.8% was obtained from Linde (Barcelona, Spain).

### 2.2. Plant Material

Olive pruning waste (500 g) was collected in March 2023 by “Olivarera San José de Lora de Estepa Cooperative” (Seville, Spain). Drying was carried out in an oven at 40 °C for one day. The pruning waste was crushed in an electric grinder for a period of 1.5 min to avoid thermal degradation, and sieved to select an average particle diameter of around 1 mm. The material was stored at room temperature.

### 2.3. Preparation of Pruning Olive Leaf Extract

**Figure 1:**
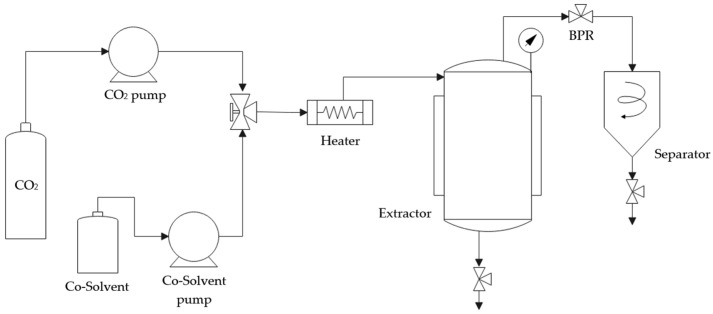
Scheme of SF100 pilot plant.

The extraction of antioxidant compounds from olive pruning waste was performed by enhanced solvent extraction. The best conditions of pressure (120 bar), temperature (80 °C) and CO_2_ flow rate (10 g/min) from previous studies were used [28]. The extraction was performed for 24 h in an SF100 pilot plant developed by Thar Technologies (Pittsburgh, PA, USA). This plant, which is shown in Figure 1, included an extraction vessel of 1 L, two high-pressure pumps for co-solvent (ethanol) and CO_2_, a back-pressure regulator to control system pressure and a separator at the end of the line.

An approximate quantity of 120 g of triturated olive pruning waste and 650 mL of ethanol were introduced into a 1 L-vessel, and an enhanced solvent extraction (ESE) was carried out according to the method previously described by Chinnarasu et al. [29], using CO_2_ in combination with ethanol as co-solvent. The ESE extraction was carried out with a 50% CO_2_–50% ethanol ratio. Thus, the CO_2_ increased the diffusion and the ethanol improved the extraction of polyphenols. The final concentration of the extract was 106.2 mg dry weight/mL ethanol.

### 2.4. Supercritical Antisolvent Extraction (SAE)

**Figure 2:**
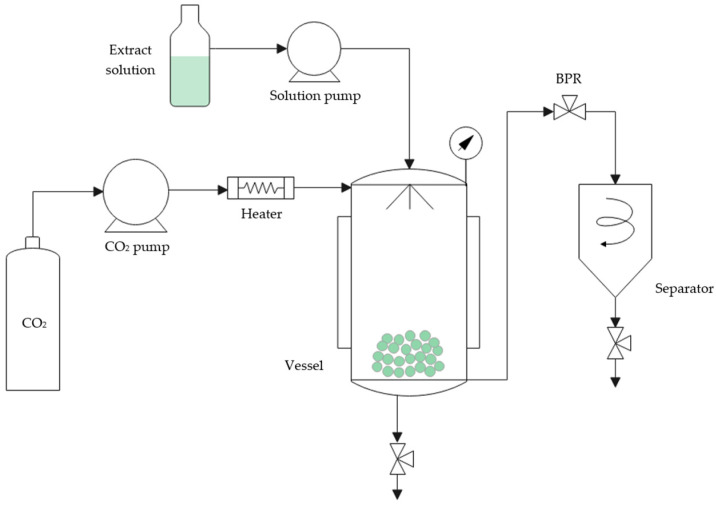
Scheme of SAS Iberfluid pilot plant.

The SAE technique was used to precipitate particles from olive pruning waste. A pilot plant built by Iberfluid (Barcelona, Spain) (SAS) was used. A schematic diagram of the pilot plant is shown in Figure 2. The equipment included two high-pressure pumps, one for the CO_2_ and the other one for the extract solution; a precipitator vessel (0.5 L); an automated high-precision back-pressure regulator (BPR) attached to a motor controller with a position indicator; and a separator (0.5 L). Two pressure gauges were included in the precipitator and separator vessel. Finally, a manual back-pressure regulator at the end of the line was required to achieve the separation of solvent and CO_2_ once the depressurization was made.

A factorial design of experiments of mixtures of level 3 × 2^2^ was employed. In this design, pressure and temperature variables had two levels, and the extract–polymer ratio (*w*/*w*) had three levels, because the extract–polymer ratio could be a key parameter in the encapsulation process. The other selected conditions—CO_2_ flow rate (30 g/min), solution flow rate (6 mL/min), concentration of solution (20 mg/mL), nozzle diameter (100 μm) and washing time (60 min)—were selected according to previous work and held constant [20]. The conditions of the fourteen experiments are described in Table 1.

### 2.5. Particle Size Distribution

The morphology and size of the samples were evaluated using a Nova NanoSEMTM 450 scanning electron microscope (SEM). The precipitated particles were previously covered with a coating of 15 nm film of gold using a sputter coater. SEM images were processed using Scion image 4.0 analysis software (Scion Corporation, Chicago, IL, USA) to obtain the particle sizes. The software Statgraphics Centurion XIX (version 19.6.03) was used to calculate the mean particle size and particle size distribution.

### 2.6. Antioxidant Activity

Antioxidant activity was determined using the DPPH assay (2,2-Diphenyl-1-picrilhydrazole) described by Scherer and Godoy [30]. The radical DPPH is a soluble and stable molecule in methanol that is characterized by its intense violet color with a maximum absorption at 515 nm. Antioxidants react with this stable radical by providing it with an electron or hydrogen atom, thereby reducing it to 2,2-diphenyl-1-hydrazine (DPPH-H) or substituted analog hydrazine (DPPH-R) characterized by colorlessness or a pale yellow color, which is then measured by spectrophotometry.

In order to perform the test, a calibration line was used, in which Equation (1) was obtained. The line was made from different concentrations of DPPH, ranging from 31.25 μg/mL to 2000 μg/mL. They were obtained from successive dilutions by taking an 8 × 10^−5^ mol/L stock solution of DPPH in ethanol preserved at low temperatures and in darkness.

(1) Abs=11079·concentration−0.0278

First, a 6 × 10^−4^ M concentration DPPH stock solution was prepared. To do this, 11.83 mg of the commercial powdered reagent was taken and diluted with 50 mL of ethanol, thus obtaining our desired concentration. The test required a dilution of the stock solution with a concentration of 6 × 10^−5^ M. For this purpose, a dilution of 1:10 *v*/*v*. was made. Once prepared, the dilution of DPPH was stored in the freezer and in darkness. Dilutions were then made of the 14 samples of the particles obtained. From each sample, 10 wells were loaded in the multiplaca at different concentrations, performing their respective dilutions.

Then, 2 h of incubation was left in the absence of light, optimal time for the reaction to occur completely in the case of antioxidants present in olive leaves. The absorbance was measured after that time using the “Synergy HTX multi-mode reader” equipment. The program “Gen5 3.12” was used to display the results. From the absorbances obtained and the calibration line, the antioxidant capacity of the particles was obtained, which is expressed by calculating the antioxidant activity index (AAI) (Equation (2)):

(2) AAI=DPPHf(μgmL)EC50(μgmL)

For the evaluation of the antioxidant activity, the EC50 parameter was also used, which refers to the concentration required to reduce the 50% of DPPH present at the beginning. It is a parameter used to express the antioxidant capacity and to compare the activity of different composites. In this paper, the determination of the antioxidant capacity of particles is based on the AAI values described by Scherer and Godoy.

### 2.7. Total Phenolic Content

The total phenolic content (TPC) of the particles obtained was determined using the Folin–Ciocalteu method proposed by Singleton et al. [31] with several modifications for microplates [32]. It is based on a suitable analytical technique with good reproducibility for the determination of total polyphenols in biological materials. The content of phenolic compounds is determined based on the reduction of Mo^6+^ to Mo^5+^, which is blue. The mixture of Folin–Ciocalteu reagent and phenolic compounds is stable in acid but unstable in alkaline solution. Therefore, sodium carbonate is used to provide an alkaline environment, essential for the reaction. The intensity of the blue colour is proportional to the amount of phenolic compounds present in the sample. To determine the phenolic content, a calibration curve using gallic acid as standard was used and the absorbance of the sample at 725 nm was determined. An aliquot of 12.5 μL of each extract dissolved with ethanol at 10,000 μg/mL was taken to perform the method. It was then mixed with 12.5 μL of Folin–Ciocalteu reagent and 200 μL of double-distilled water. Once added, these 3 components were shaken for 5 min. Then, 25 μL of sodium carbonate solution (20% *w*/*v*) was added and stirred again for 5 min. Then the 60 min multiplex was left in the dark at room temperature and the absorbance was measured at 725 nm using the “Synergy HTX multi-mode reader” equipment. The program “Gen5 3.12” was used to visualize the results. To construct the calibration line, dilutions of gallic acid of concentrations from 0.39 μg/mL to 25 μg/mL were used to obtain the equilibrium line with its corresponding equation (Equation (3)) to obtain the concentration of phenolic compounds from the absorbances obtained:

(3) Absorbance=0.07·concentration−0.0247

The obtained results were expressed in µg gallic acid equivalents per gram of encapsulated particles, as shown in Table 1.

### 2.8. Residual Solvent

Residual ethanol (RE) in the particles was assessed by measuring the loss of weight after heating a known quantity of sample (0.05 g) at a temperature of 40 °C for 24 h. The RE was determined using Equation (4) and the results were reported as a mass percentage (% *w*/*w*), representing grams of residual solvent per 100 g of dry co-precipitate.

(4) $$Residual\;ethanol=\frac{{Mass}_{\;initial\;particles}-{Mass}_{\;final\;particles}}{{Mass}_{\;initial\;particles}}\times 100$$

### 2.9. Controlled Release Test in Simulated Fluids

The precipitated extract without the polymer and the co-precipitates of runs 1, 5, 7 and 12 (experimental parameters in which encapsulated spherical particles were obtained, and sample 12, where the highest antioxidant capacity was obtained) were subjected to in vitro controlled release in two types of simulated gastrointestinal fluids. Simulated gastric fluid with a pH of 1.2 was prepared by dissolving 2 g of sodium chloride in 1 L of water and adding 0.2 N HCl to adjust the final pH. Intestinal simulated fluid was prepared by dissolving 6.8 g of monobasic phosphate in 1 L of water and adjusting the pH to 6.8 with 0.2 N NaOH.

A similar procedure was followed for each simulated fluid. In 40 mL of simulated liquid, 10 mg of each co-precipitate sample was mixed. The tests were carried out with continuous stirring at a temperature of 37 °C. At each predetermined time (5, 15, 30, 60, 120, 240 and 480 min), 2.5 mL aliquots were taken and replaced with 2.5 mL of fresh simulated fluid. The aliquots were filtered and their absorbances were measured using a Shimadzu UV. The absorbance was measured at λ = 660 nm, which is the wavelength corresponding to the highest olive leaf extract absorbance without the interference of any components produced by the degradation of the polymer. The correction proposed by Zhu et al. [33] was used to correct the concentration of extract released.

## 3. Results and Discussion

**Figure 3:**
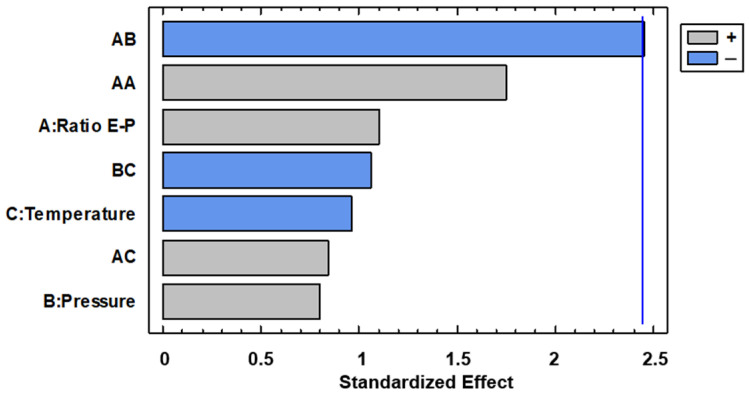
Pareto diagram for total collected mass of particles.

**Figure 4:**
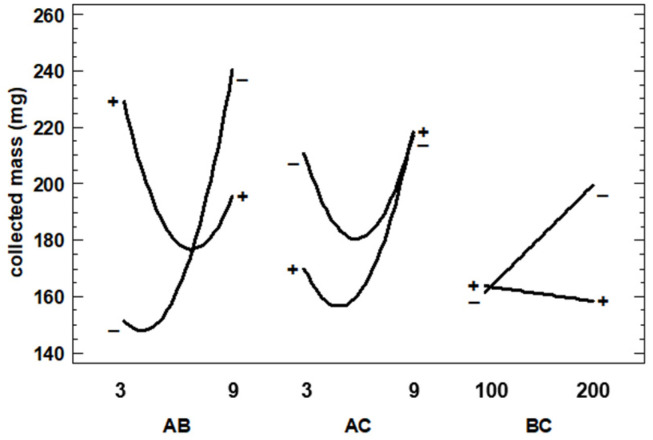
Interaction plots for collected mass of particles (A: E–P ratio; B: Pressure; C: Temperature).

Precipitation of the extracts was achieved in all the cases. The powder was collected and weighed, ranging between 125.0 (run 14) and 252.6 mg (run 2) of the collected mass, as can be seen in Table 1. The Pareto diagram shown in Figure 3 indicates that the Extract–Polymer ratio × Pressure interaction is the only significant effect. The interaction plots are shown in Figure 4.

In this Figure, it can be seen how the behavior of one variable is dependent on whether another one changes from its low to its high value. In this sense, it could be found that the variable is independent of another variable or even have an inversion of the trend. In the case of Extract–Polymer ratio × Pressure (AB), according to Figure 4, at a low ratio (1:3), the collected mass increases when pressure is increased, but at a high ratio (1:9), the collected mass increases when pressure is decreased.

**Figure 5:**
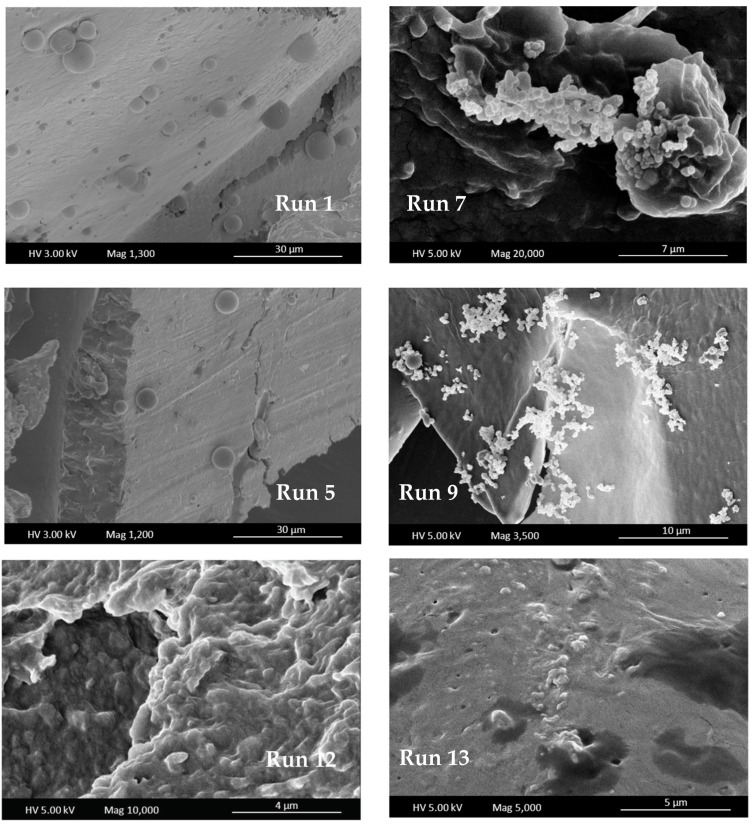
SEM images of precipitated particles (runs 1, 5, 7, 9, 12 and 13).

On the other hand, the morphology of the particles was different depending on the operating conditions. In Figure 5, examples of different situations are shown: extracted spherical particles encapsulated by the polymer that was immersed, in turn, in a polymer matrix (run 1), or precipitated extracts on the polymer surface (runs 7 and 14) or even extracts precipitated totally covered by a layer of polymer (run 13). In this sense, only the particles corresponding to runs 1, 5, 7, 8, 9 and 14 with mean particle size from 0.3 ± 0.9 to 4.6 ± 3.9 µm could be measured. 

This difference in diameter could be explained due to the encapsulation or co-precipitation of the particles. In the case of co-precipitation, the smaller particles would correspond to pruning waste extract on the polymer surface, but when the particles are encapsulated, larger particles are formed.

**Figure 6:**
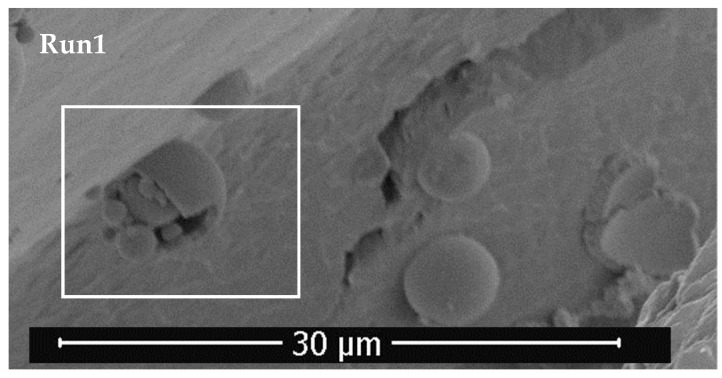
Zoom of SEM images of run 1.

In Figure 6 can be seen a broken polymer sphere containing smaller spheres of pruning waste extract. As can be observed in SEM images, these spheres that are encapsulated are also immersed in a layer of polymer. Thus, the difference in size of precipitated particles lies in whether the process achieves encapsulation or if precipitated particles of extract remain on the polymer surface. Olive leaf particles were precipitated in previous work [29] in the submicron range, similar to runs 7–9, and when the polymer encapsulation happens, the precipitated particles increased their size to 5–7 µm. The influence of operating conditions on particle size cannot be described, mostly due to experiments not resulting in precipitation in the form of particles.

On the other hand, antioxidant activity could be measured in most experiments; the results are shown in Table 1. Precipitates from runs 1, 2, 6 and 7 had moderate antioxidant activity, and run 14 had a higher value, considered as strong antioxidant activity. Anyway, it should be taken into account that the antioxidant test is done with composites based on polymer and extract, and thus the antioxidant capacity would correspond to a part of the weighted sample, so the data are undervalued. In this sense, in the 1:6 ratio, values of 0.5–0.6 were observed, except for run 12, with the highest antioxidant activity. If the polymer ratio is the same in run 12, it will have precipitated compounds with higher antioxidant activity than the rest. 

**Figure 7:**
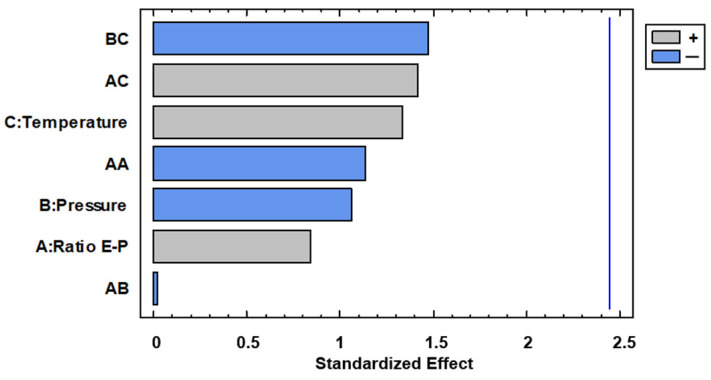
Pareto diagram for antioxidant activity.

Thus, higher pressure and especially higher temperatures are recommended to generate strong antioxidant particles. When a 1:9 ratio is used, 0.7–0.8 of AAI is observed. This means that in spite of these runs having the higher polymer content, they showed a moderate antioxidant activity, and thus the precipitated compounds would be quite antioxidant. With regard to experiments where the lower ratio 1:3 is used, a lower AAI was observed, as expected, since the composites would be formed by a higher amount of extract. Anyway, from the Pareto diagram (Figure 7), the analyzed variables seem to be not significant on AAI.

**Figure 8:**
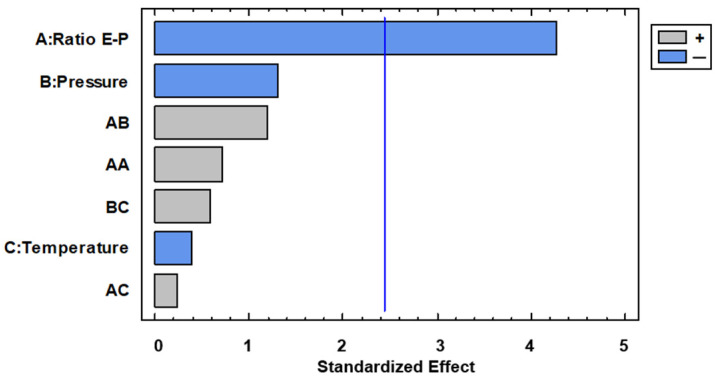
Pareto diagram for total polyphenol content.

With regard to total polyphenol content, it can be observed in the Pareto diagram of Figure 8 that Extract–Polymer ratio was the only variable with significance.

**Figure 9:**
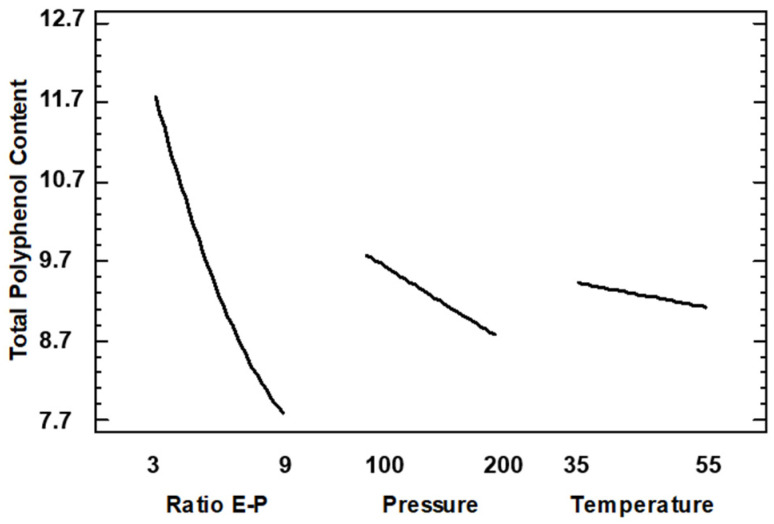
Main effect of variables on total polyphenol content. Results expressed as mg GAE/g particles.

**Figure 10:**
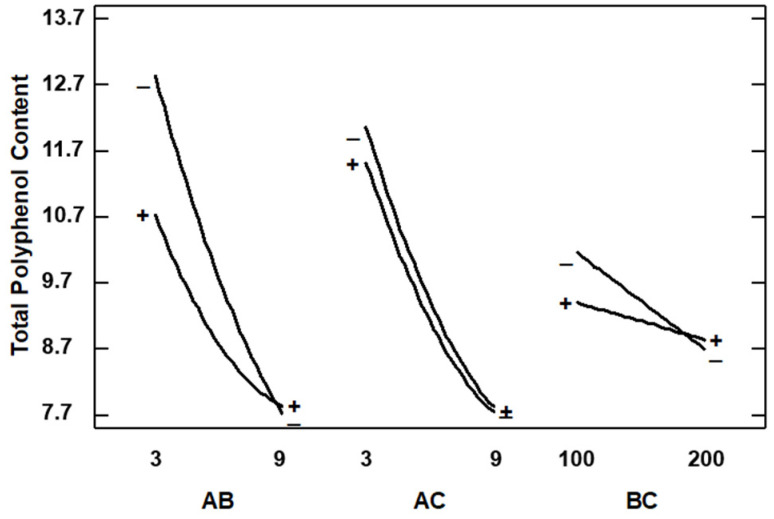
Interaction plots for total polyphenol content (A: E–P ratio; B: Pressure; C: Temperature). Results expressed as mg GAE/g particles.

In Figure 9 is shown the main effect on TPC, and it can be observed that effectively the curve of the Extract–Polymer ratio has a high slope, indicating the strong influence on TPC. Thus, a lower Extract–Polymer ratio is recommended to increase the amount of polyphenol in the composites. This trend is expected due to the composites being formed by a smaller amount of polymer. Thus, these data do not agree with AAI since it would be expected that the higher the AAI, the higher the TPC, and this showed the opposite trend: the higher the TPC, the lower the AAI. We analyzed the interaction plots shown in Figure 10, and lower ratios and lower pressures are recommended to increase the amount of polyphenols in the sample, but at this ratio, temperature does not have any influence. This discrepancy between the data on antioxidant activity and total phenolic content in the encapsulates can be explained by several reasons. Firstly, the composition of both the initial extract and the capsules is extremely complex in terms of existing compounds. The fact that the SAE technique generally concentrates the particles on certain compounds does not mean that, due to the multitude of other compounds, the final antioxidant activity changes as the total phenolic content changes [34]. On the other hand, according to another possible explanation, other authors confirm that under certain conditions, antioxidants can act as pro-oxidant species related to their redox potential and the concentration of these antioxidants in the matrix environments [35]. In general, due to the high complexity of the extract in terms of compounds present, further optimization studies on the selectivity of the process are necessary.

Regarding the residual ethanol remaining in the samples after the supercritical precipitation process, the results can be observed in Table 1, expressed in % *w*/*w*. There is a clear trend towards less residual solvent when the pressure used in the process is higher (*p*-value = 0.035). On the other hand, both the Extract–Polymer ratio and temperature had no significant effect on solvent removal from the co-precipitates. This significant negative effect of pressure could be explained by the fact that the drying mechanism of the spray produced during the SAE process is mostly controlled by the transfer of matter between the supercritical phase and the microdroplets that are formed, the heat transfer mechanism being less important (evaporation) [36]. Pressure increases in the system at constant temperature tend to cause an increase in the diffusion of pressurized CO_2_ into the microdroplets, as well as an increase in the solubility of the solvent in CO_2_. This usually causes a decrease in the solvating power of the solvent, which leads to a higher degree of supersaturation, resulting in less organic solvent in the final samples. This is usually accompanied by an improved particle size, but this tendency is not observed in the results previously shown.

Similar results have been obtained in the literature regarding the residual solvent present in the particles using the antisolvent effect of supercritical CO_2_. Majerik at al. obtained oxeglitazar particles encapsulated with different polymers, where residual concentrations between 3 and 5% were reported [37]. Zabot and Meireles, precipitating particles rich in quercetin and other polyphenols, obtained results around 1.5–2.0% residual solvent [38]. Compared to other techniques, spray-drying (2%) or freeze-drying (1%) [39], the obtained results are still similar, and in some cases, better. This indicates that, under specific conditions of pressure and temperature, the existing SAE process demonstrates comparable efficiency to other particle formation/drying methods in the removal of the residual solvent.

**Figure 11:**
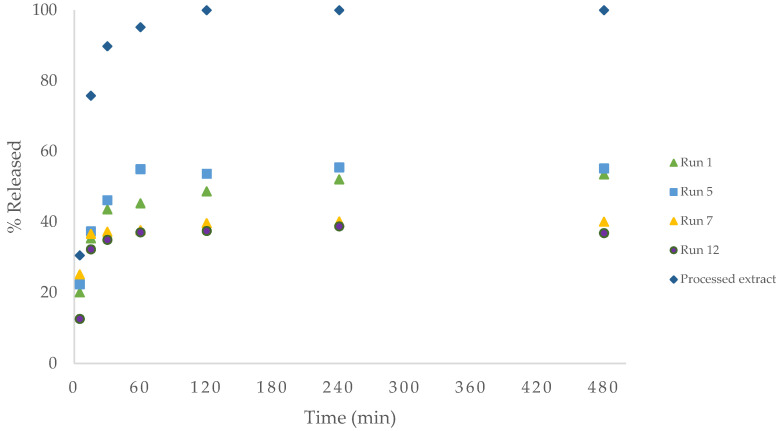
Release profile of total extract in simulated gastric fluid, pH 1.2.

**Figure 12:**
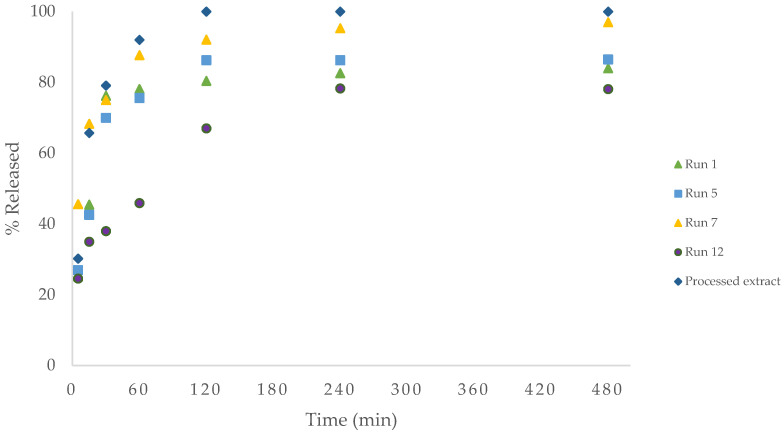
Release profile of total extract in simulated intestinal fluid, pH 6.8.

Controlled release in simulated fluids of different pH was performed on the samples that showed the highest antioxidant activity, as the most promising for potential drug treatments. Prolonged gastric retention not only improves bioavailability but also mitigates drug waste, concurrently enhancing the solubility of medications that exhibit limited solubility in a high-pH environment [40]. The main objective of encapsulation in this work was to develop a drug delivery system which can reside in the stomach for longer time and release the bioactive compound of the extract for as long as possible in the intestinal fluids, where there is the highest population of bacteria. Release in gastric fluid and intestinal fluid, as shown in Figure 11 and Figure 12, respectively, of experiments 1, 5, 7, 12 and the unencapsulated precipitated extract (100 bar, 40 °C), was performed to compare their behaviour.

In general, the release of the unencapsulated extract particles is faster than that of the drug–polymer systems in both fluids. As can be observed in Figure 11, there is a lower release of the extract in the encapsulated samples, possibly due to the fact that Pluronic F-127 has low solubility in acidic medium [41,42]. Additionally, the samples experienced a rapid burst release during the initial stage, likely due to the rapid release of surface-bound drug molecules. Other authors argue that the rapid initial release in drug–polymer systems is due to diffusion of the drug through the matrix [43]. Thus, the rest of the compounds remain immobilized within the crystalline domains and are released only when the matrix degrades to a higher pH in this specific case. It is worth noting that the lowest release of the compounds was in run 12, where it is possible that the extract precipitated totally, covered by a layer of Pluronic, as its particle size could not be measured due to its low homogeneity.

Regarding the release at pH 6.8 of simulated intestinal fluid (Figure 12), as expected, the unencapsulated particles were released more rapidly, releasing about 90% in the first hour. In comparison to the results obtained with gastric fluid, the amount of bioactive compounds released is significantly higher, achieving releases above 80% in all cases. This fact is due to the already-mentioned superior solubility of both the polymer and the polyphenols in a neutral medium.

Finally, neither the pressure nor the temperature used seems to have a clear influence on the rate of release of the compounds nor on the total amount released. It is true that in the case of gastric fluid, a higher amount of release was obtained in the test with the lower polymer used, run 5, which makes sense because of the possible lower degree of encapsulation. However, this was not observed in the intestinal fluid. This can be explained by the aforementioned presence of extract on the surface of the polymer, causing a burst release. This behaviour has been described in previous work using a pure antioxidant such as mangiferin in the same medium, typically present in most natural ethanol extracts from leaves or prunings [44].

## 4. Conclusions

For the system formed by the olive pruning residue extract and the encapsulating polymer Pluronic F127, the pressurized antisolvent process under supercritical CO_2_ conditions proved to be effective and promising for obtaining sub-micro particles with a relatively high antioxidant power, rich in polyphenols. Through the SAE process, it was possible to precipitate particles with moderate antioxidant activity, exceeding 0.5 AAI in most cases, with a high polyphenol content (up to 13 mg GAE/g of particles). The extract–polymer ratio demonstrated a significant negative effect on the final polyphenol concentration, while increases in pressure resulted in particles with a lower percentage of residual organic solvent. Particle sizes ranging from 0.3 to 4.6 µm were obtained, but it was impossible to determine the influence of operational variables since many of the experiments did not result in uniform particles. Regarding the controlled–release study, an enhancement was observed compared to non-encapsulated extract particles, with delayed releases in gastric fluid and enhanced release in intestinal fluid. This shows the significant potential of the proposed process in pharmaceutical or food sector applications. However, although successful under certain conditions, further studies are recommended to optimize the samples, reducing the particle size to the nanometer range, to ensure a uniform particle size distribution and to minimize polymer agglomeration.

## Figures and Tables

**Table 1:** Design of SAE experiments and results.

Run	Ratio Extract–Polymer ^1^	P (Bar)	T (°C)	PS ^2^ (µm)	Collection (mg)	AAI ^3^	TPC ^4^ (mg GAE/g)	RE ^5^ (*w*/*w* %)
1	1:6	150	47	4.6 ± 3.9	205.3	0.6	7.9 ± 0.5	0.7 ± 1.2
2	1:9	100	55	---	252.6	0.8	6.9 ± 1.3	3.2 ± 0.5
3	1:3	200	40	---	248.6	0.3	10.5 ± 3.1	0.4 ± 1.2
4	1:6	200	40	---	210.6	0.5	9.8 ± 2.6	0.7 ± 0.1
5	1:3	100	55	3.7 ± 2.2	151.6	0.3	12.5 ± 3.6	2.3 ± 3.4
6	1:9	200	55	---	196.1	0.7	8.9 ± 0.5	0.6 ± 0.7
7	1:6	100	40	0.3 ± 0.9	132.1	0.6	9.4 ± 2.2	3.3 ± 2.1
8	1:9	200	40	0.4 ± 0.2	185.2	---	6.7 ± 1.1	0.2 ± 0.5
9	1:9	100	40	0.5 ± 0.2	237.5	---	8.7 ± 1.3	4.5 ± 1.9
10	1:3	100	40	---	161.1	---	13.2 ± 1.2	2.7 ± 0.4
11	1:6	150	47	---	219.4	---	11.0 ± 0.4	1.1 ± 0.2
12	1:6	100	55	---	132.7	1.3	9.7 ± 2.6	1.2 ± 2.6
13	1:3	200	55	---	199.9	---	10.8 ± 4.6	0.1 ± 0.1
14	1: 6	200	55	2.5 ± 1.3	125.0	---	7.7 ± 2.3	0.4 ± 0.2

^1^ Ratio E–P expressed as (*w*/*w*); ^2^ PS = Particle size; ^3^ AAI = Antioxidant activity index; ^4^ TPC = Total phenolic content; ^5^ RE = Residual ethanol.

## Data Availability

The original contributions presented in the study are included in the article, further inquiries can be directed to the corresponding author.
